# Supplementary material for: Selective Trapping of Bacteria in Porous Media by Cell Length
Source: Integr Comp Biol. 2026 Apr 7;66:icag018. doi: 10.1093/icb/icag018 (PMC13092526; doi:10.1093/icb/icag018)
Supplement: icag018_Supplemental_Files [file icag018_supplemental_files.zip › icb-2026-0013-File012.pdf]

Supplementary Material

Selective trapping of bacteria in porous media by cell length

David Gao[1,4], Zeyuan Wang[2], Mihika Jain[3], Arnold J. T. M. Mathijssen[1], Ran Tao[1]

[1]Department of Physics & Astronomy, University of Pennsylvania, Philadelphia, PA 19104, USA

[2]Singh Center for Nanotechnology, University of Pennsylvania, Philadelphia, PA 19104, USA
[3]The Wharton School, University of Pennsylvania, Philadelphia, PA 19104, USA
[4]Department of Biology, University of Pennsylvania, Philadelphia, PA 19104, USA
*Corresponding authors: amaths@upenn.edu; rtao21@upenn.edu

Supplementary Figure S1: Distributions of straightness index and curvature in unconfined motility chambers

Supplementary Fig. S1 presents the full distributions of straightness index (SI) and trajectory curvature (κ) for cells at different induction times (0 h, 0.5 h, 1 h, and 2 h) in unconfined motility chambers. For each condition, more than 500 trajectories collected from three independent experiments were analyzed. The dashed vertical lines indicate the mean values reported in the main text (Fig. 1I). These distributions illustrate the variability underlying the mean SI and curvature values and provide the statistical basis for the SEM shown in the main figures.

Supplementary Figure S2: Straightness index distributions under ordered confinement

Supplementary Fig. S2 presents the full distributions of straightness index (SI) for cells in ordered porous environments. For each condition, more than 500 trajectories from three independent experiments were analyzed. Dashed vertical lines indicate mean SI values reported in the main text. These distributions provide the statistical basis for the SEM values shown in the main figures.

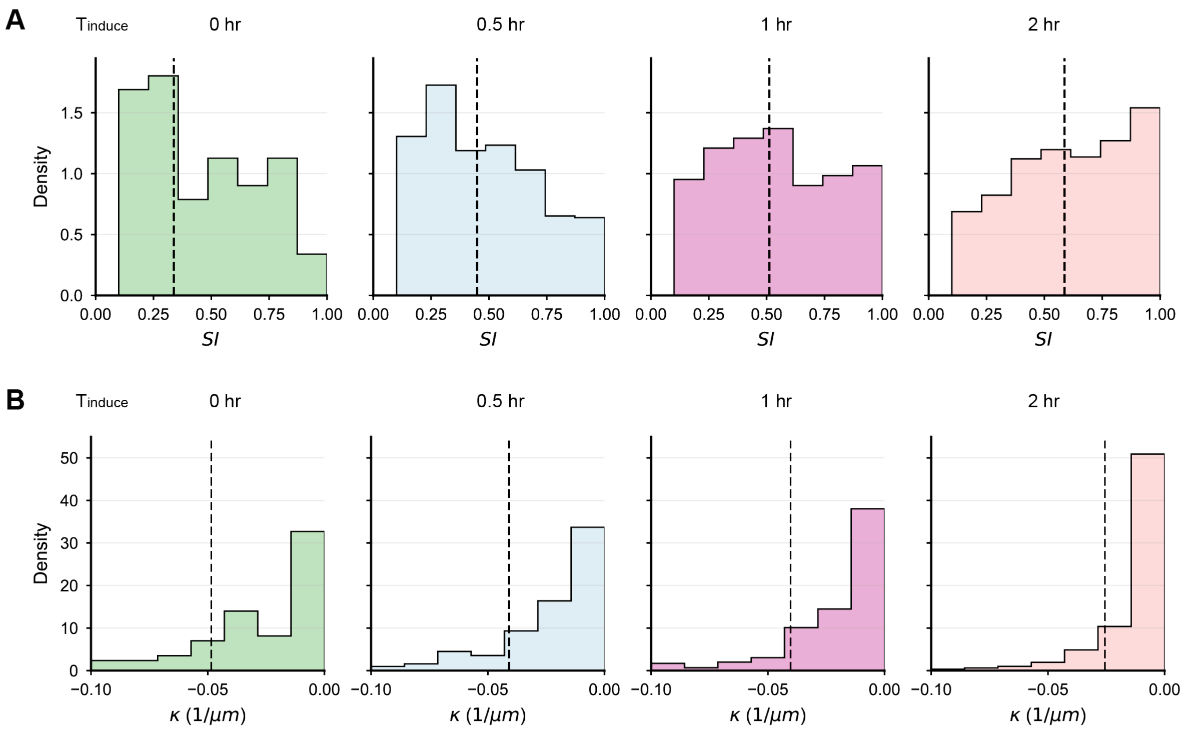

Figure S1: (A) Distributions of straightness index (SI) and (B) trajectory curvature (κ) for cells at different induction times in unconfined motility chambers. Dashed vertical lines indicate mean values. Each condition includes more than 500 trajectories from three independent experiments.

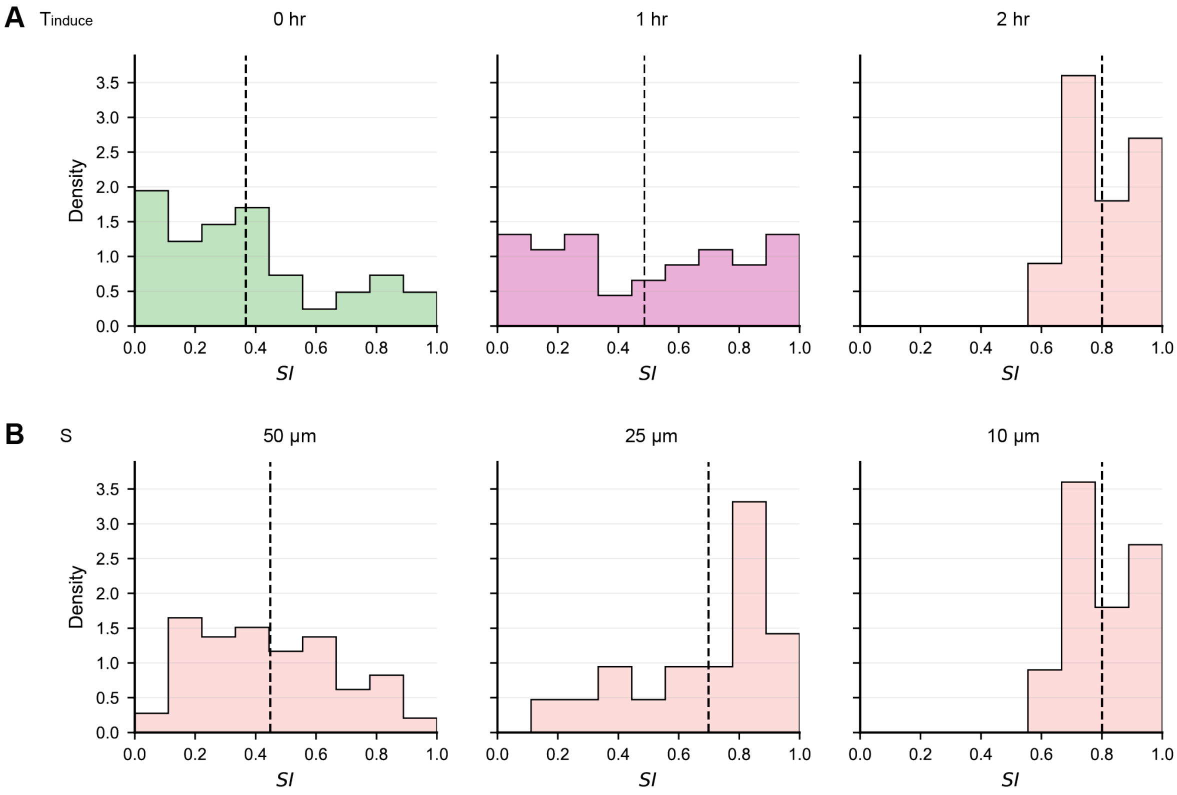

Figure S2: Straightness index (SI) distributions in ordered porous environments. (A) SI distributions for different induction times (0 h, 1 h, 2 h) at fixed geometry $R = 25\,\mu m$, $S = 10\,\mu m$. (B) SI distributions for 2 h-induced cells at different confinement spacings ($S = 50\,\mu m$, $S = 25\,\mu m$, $S = 10\,\mu m$). Dashed vertical lines indicate mean SI values. Each condition includes more than 500 trajectories from three independent experiments.
