# Supplementary figures and images for: Selective Trapping of Bacteria in Porous Media by Cell Length
Source: Integr Comp Biol. 2026 Apr 7;66:icag018. doi: 10.1093/icb/icag018 (PMC13092526; doi:10.1093/icb/icag018)

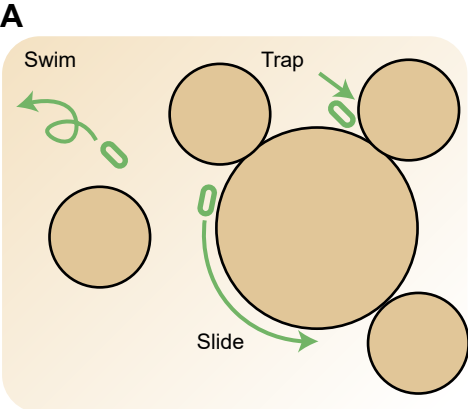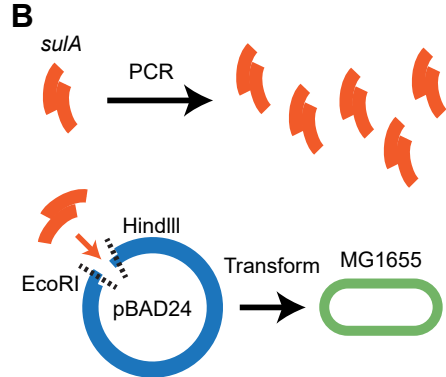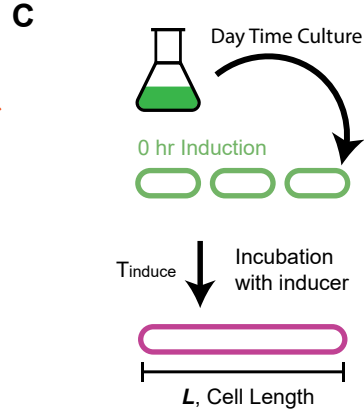

**D** 0hr Induction

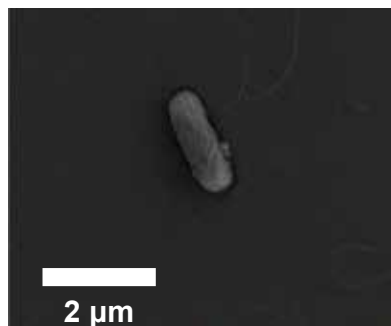

**E** 1hr Induction

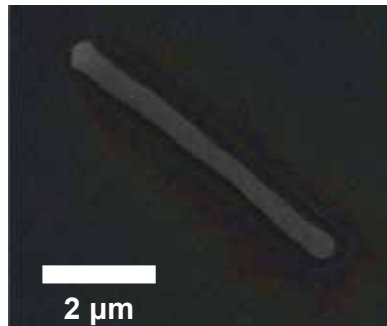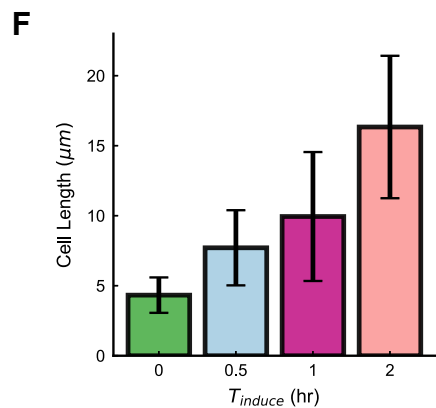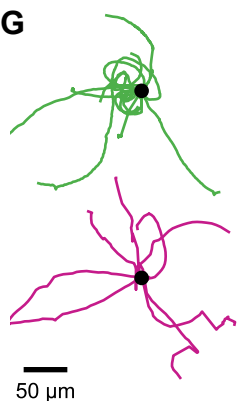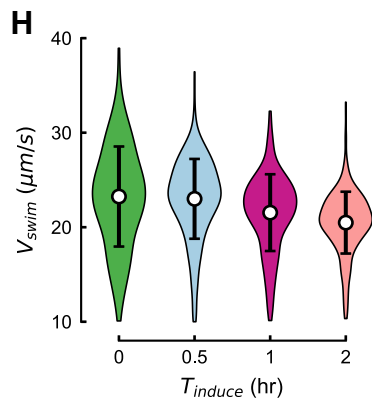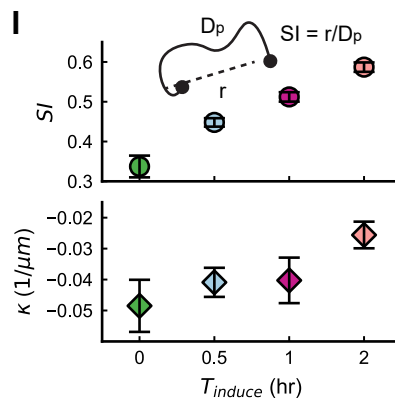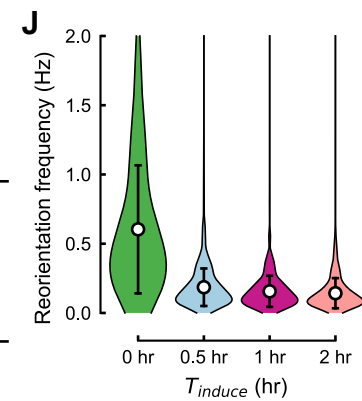

Supplement: icag018_Supplemental_Files [file icag018_supplemental_files.zip › icb-2026-0013-File007.pdf]

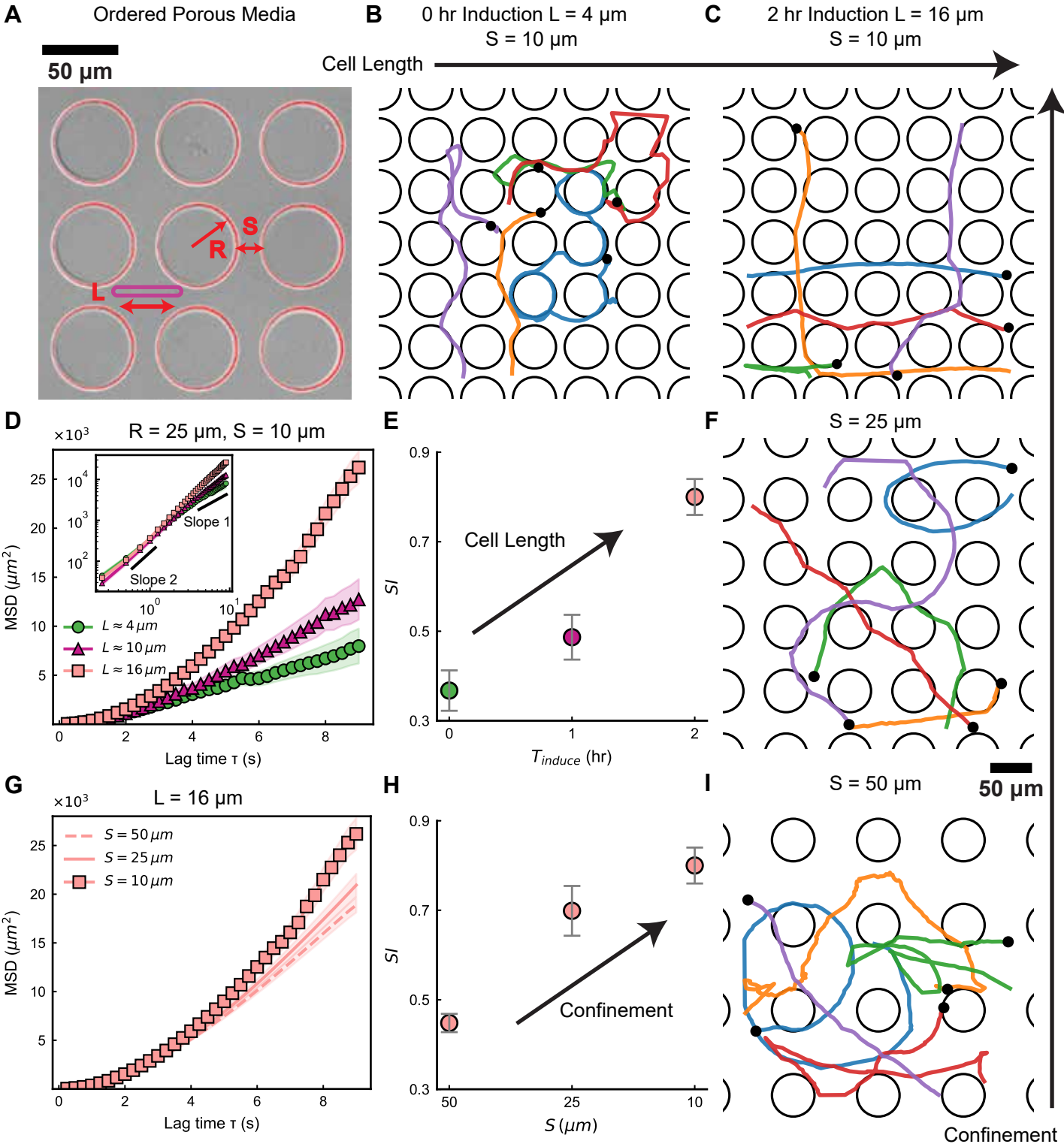

Supplement: icag018_Supplemental_Files [file icag018_supplemental_files.zip › icb-2026-0013-File008.pdf]

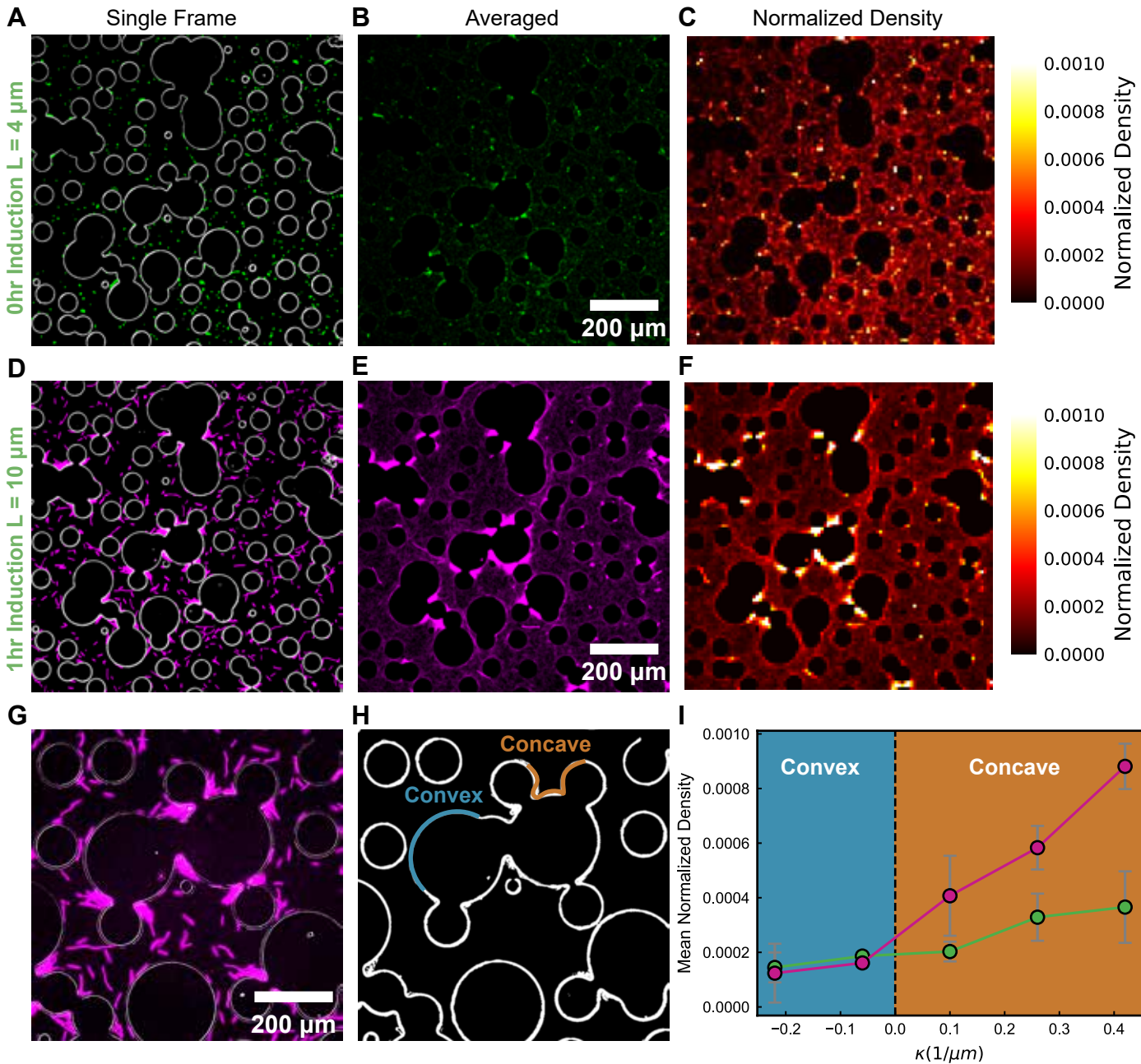

Supplement: icag018_Supplemental_Files [file icag018_supplemental_files.zip › icb-2026-0013-File009.pdf]

**A** Trap Duration in Disordered Porous Media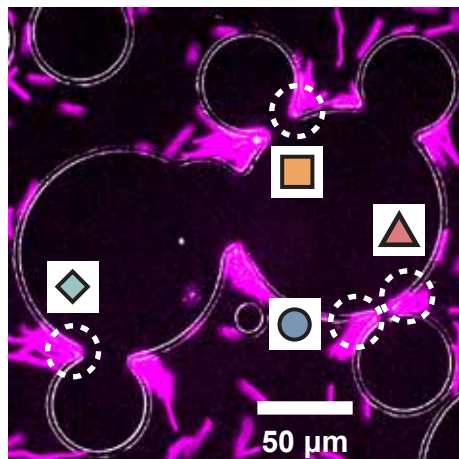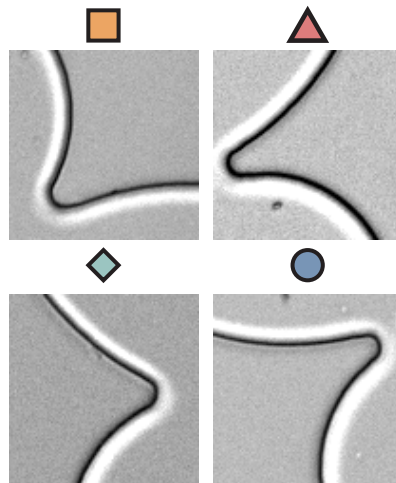**B**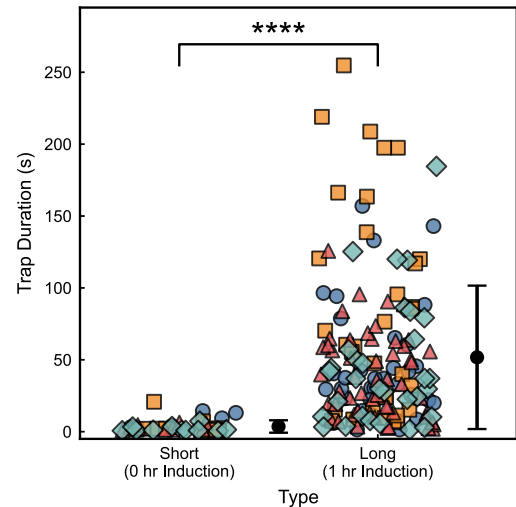**C** Short ( $L = 4 \mu\text{m}$ )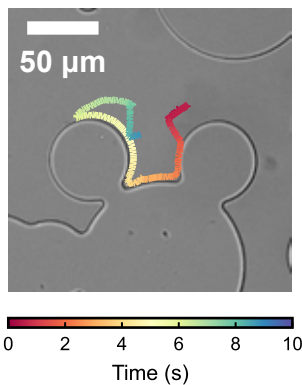**D**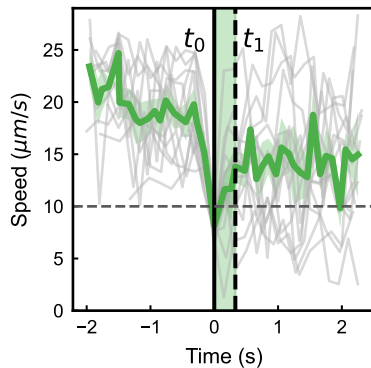**E** Long ( $L = 10 \mu\text{m}$ )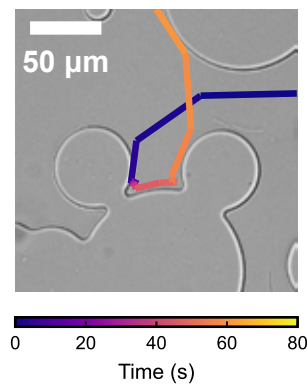**F**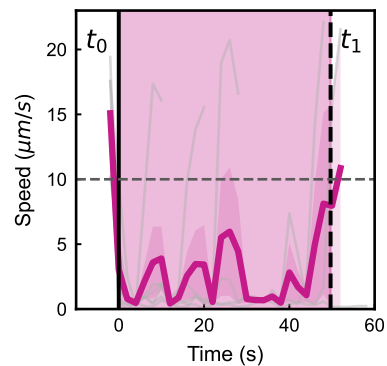

Supplement: icag018_Supplemental_Files [file icag018_supplemental_files.zip › icb-2026-0013-File010.pdf]

**A****Ordered Porous Media****Short Cells  $L = 4\ \mu\text{m}$** 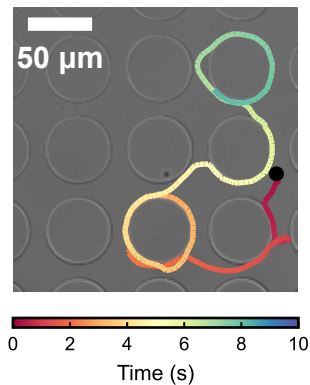**C****Disordered Porous Media**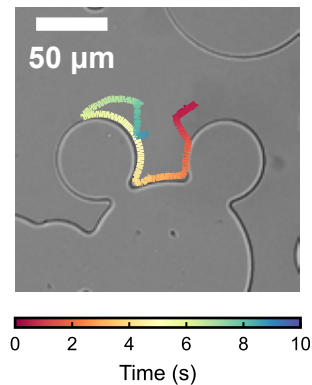**B****Long Cells  $L = 10\ \mu\text{m}$** 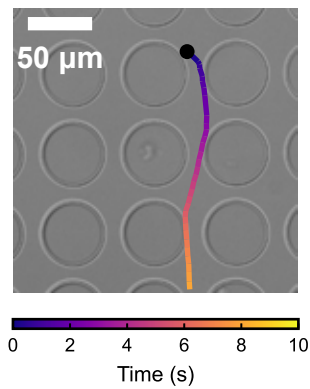**D**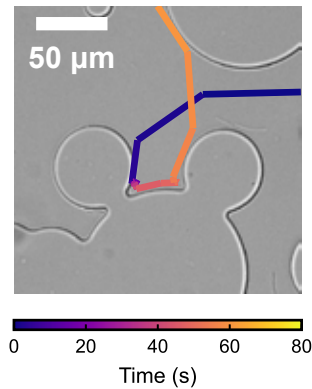

Supplement: icag018_Supplemental_Files [file icag018_supplemental_files.zip › icb-2026-0013-File011.pdf]
